# Supplementary figures and images for: Neural Selectivity for Visual Motion in Macaque Area V3A
Source: eNeuro. 2021 Jan 13;8(1):ENEURO.0383-20.2020. doi: 10.1523/ENEURO.0383-20.2020 (PMC7814481; doi:10.1523/ENEURO.0383-20.2020)

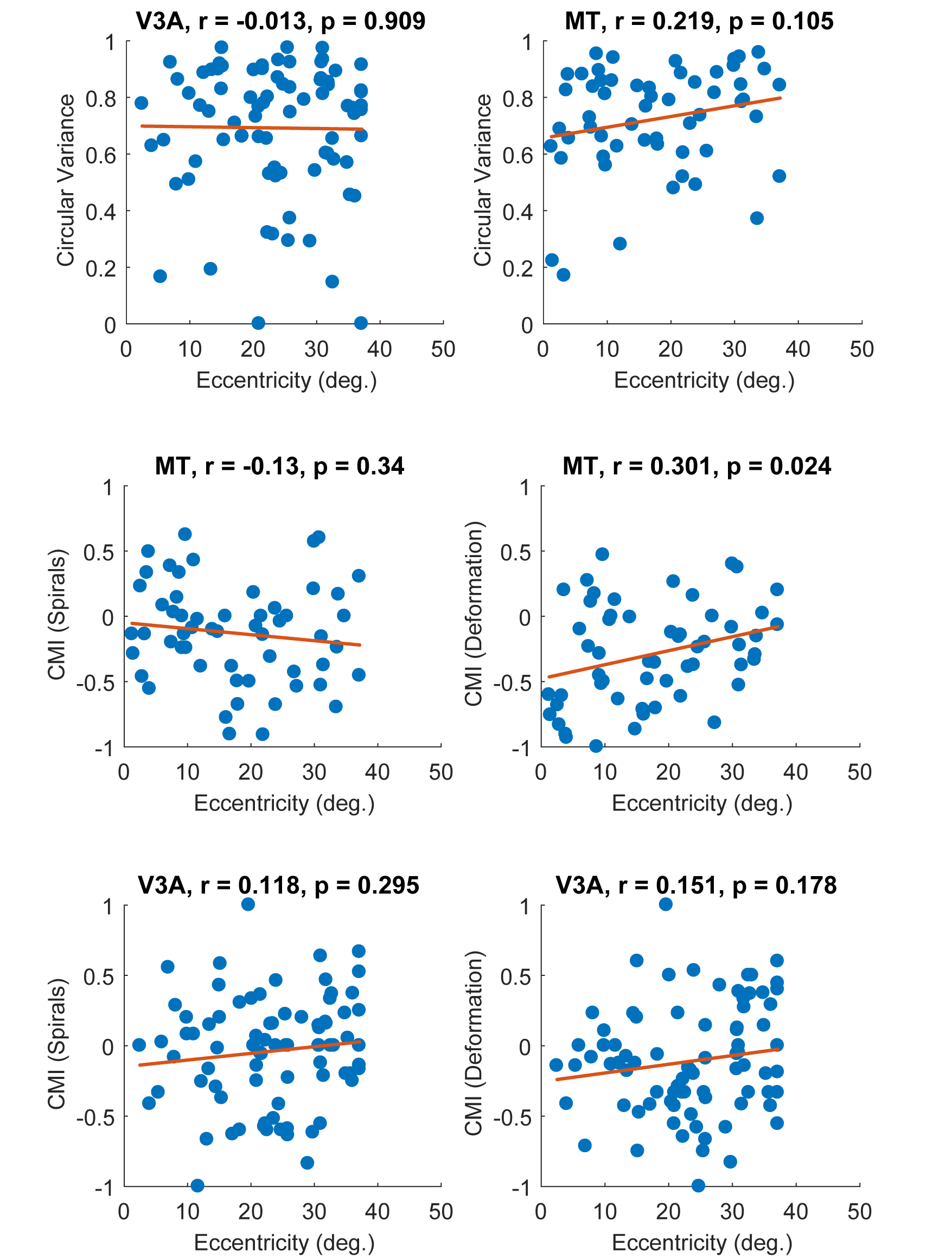

Supplement: Extended Data Figure 2-1 — Relationships of receptive field eccentricity with circular variance (upper panel), and CMI (middle and lower panel) in both V3A and MT. Each data point represents one neuron. Download Figure 2-1, TIF file. [file enu-eN-NWR-0383-20-s01.tif]

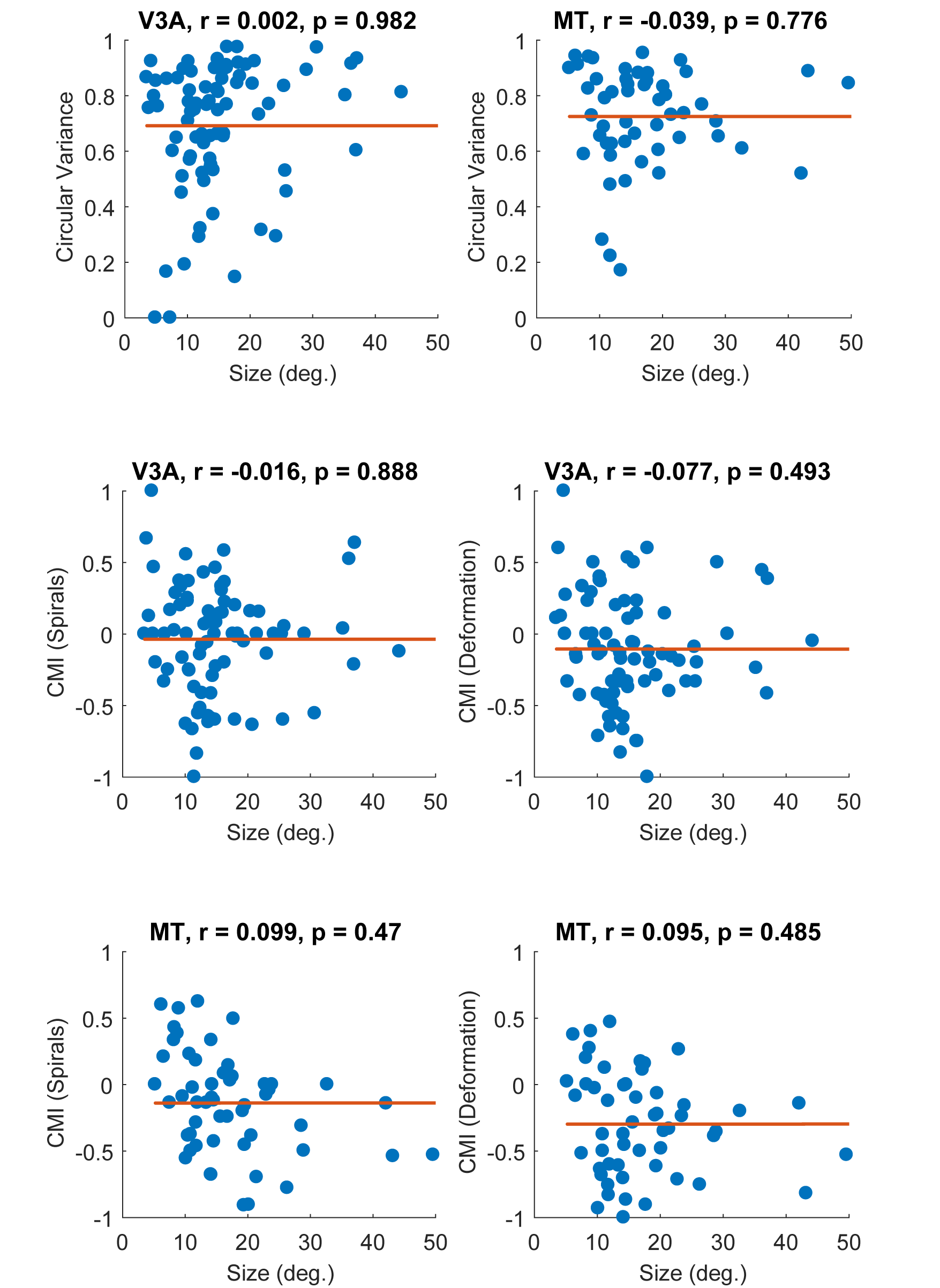

Supplement: Extended Data Figure 2-2 — Relationships of receptive field size with circular variance (upper panel), and CMI (middle and lower panel) in V3A and MT. Each data point represents one neuron. Download Figure 2-1, TIF file. [file enu-eN-NWR-0383-20-s02.tif]

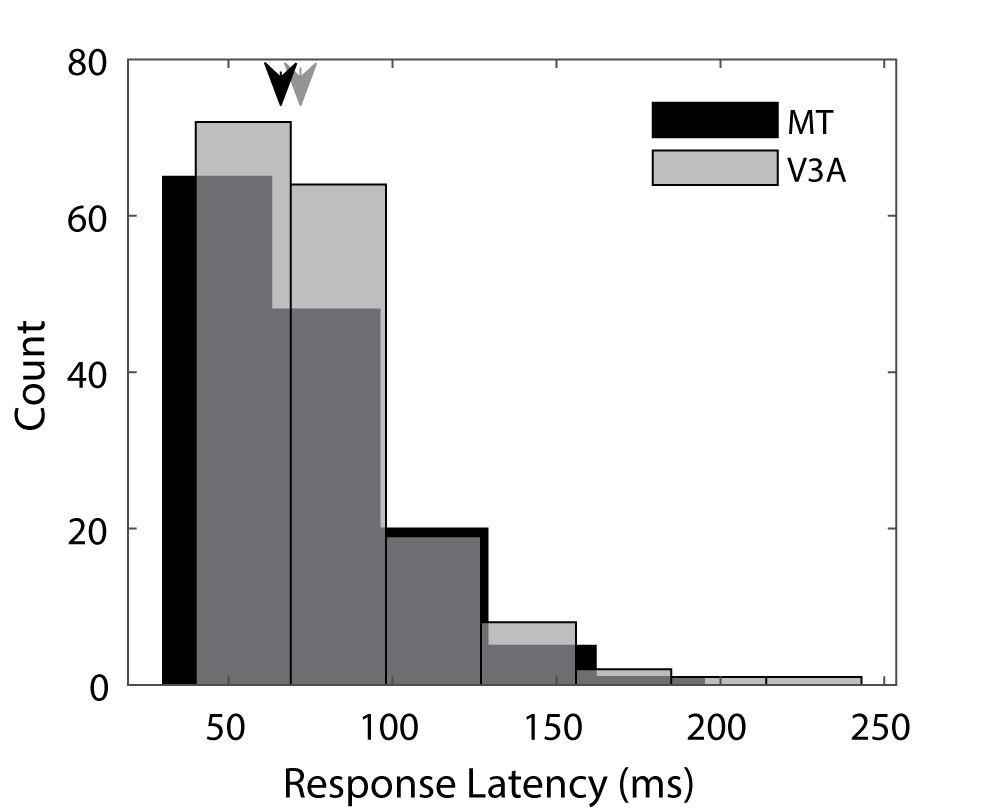

Supplement: Extended Data Figure 4-1 — Response latencies for MT and V3A. The distributions of latencies are similar between areas, although the median is slightly highger in V3A. Median values are indicated by arrows on top of the histograms; black for MT and gray for V3A. For data from each animal shown separately, please see Extended Data Figures 4-1, 4-2. Download Figure 4-1, TIF file. [file enu-eN-NWR-0383-20-s03.tif]

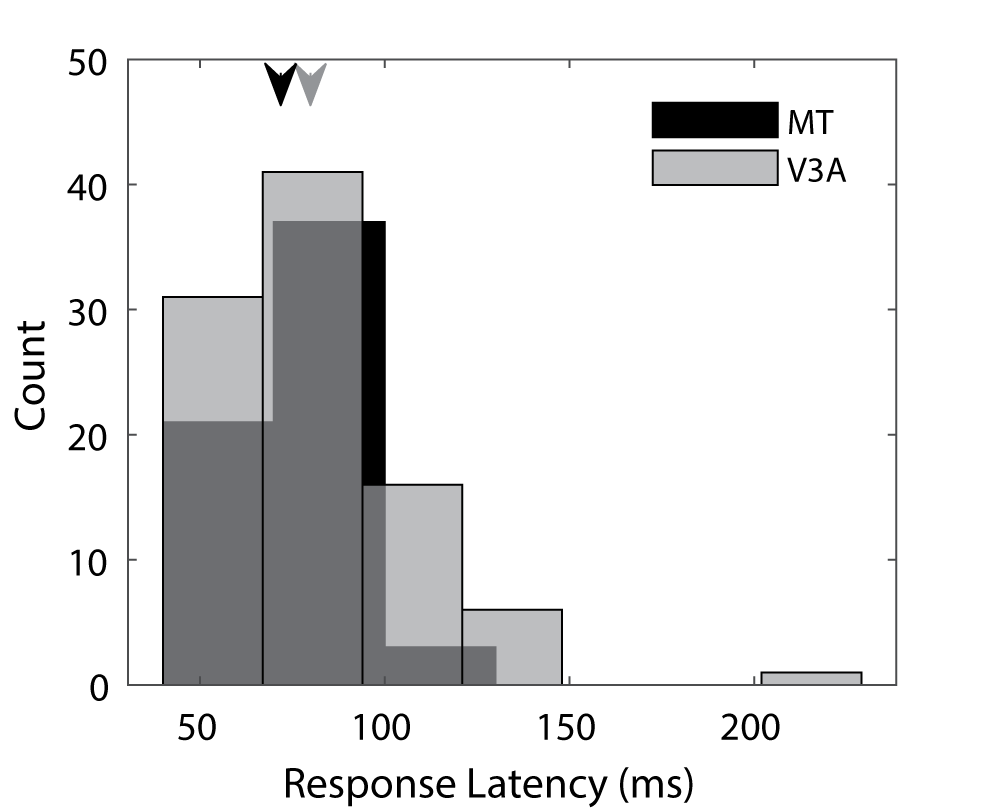

Supplement: Extended Data Figure 4-2 — Response latencies for MT and V3A. The distributions of latencies are similar between areas, although the median is slightly highger in V3A. Median values are indicated by arrows on top of the histograms; black for MT and gray for V3A. For data from each animal shown separately, please see Extended Data Figures 4-1, 4-2. Download Figure 4-2, TIF file. [file enu-eN-NWR-0383-20-s04.tif]

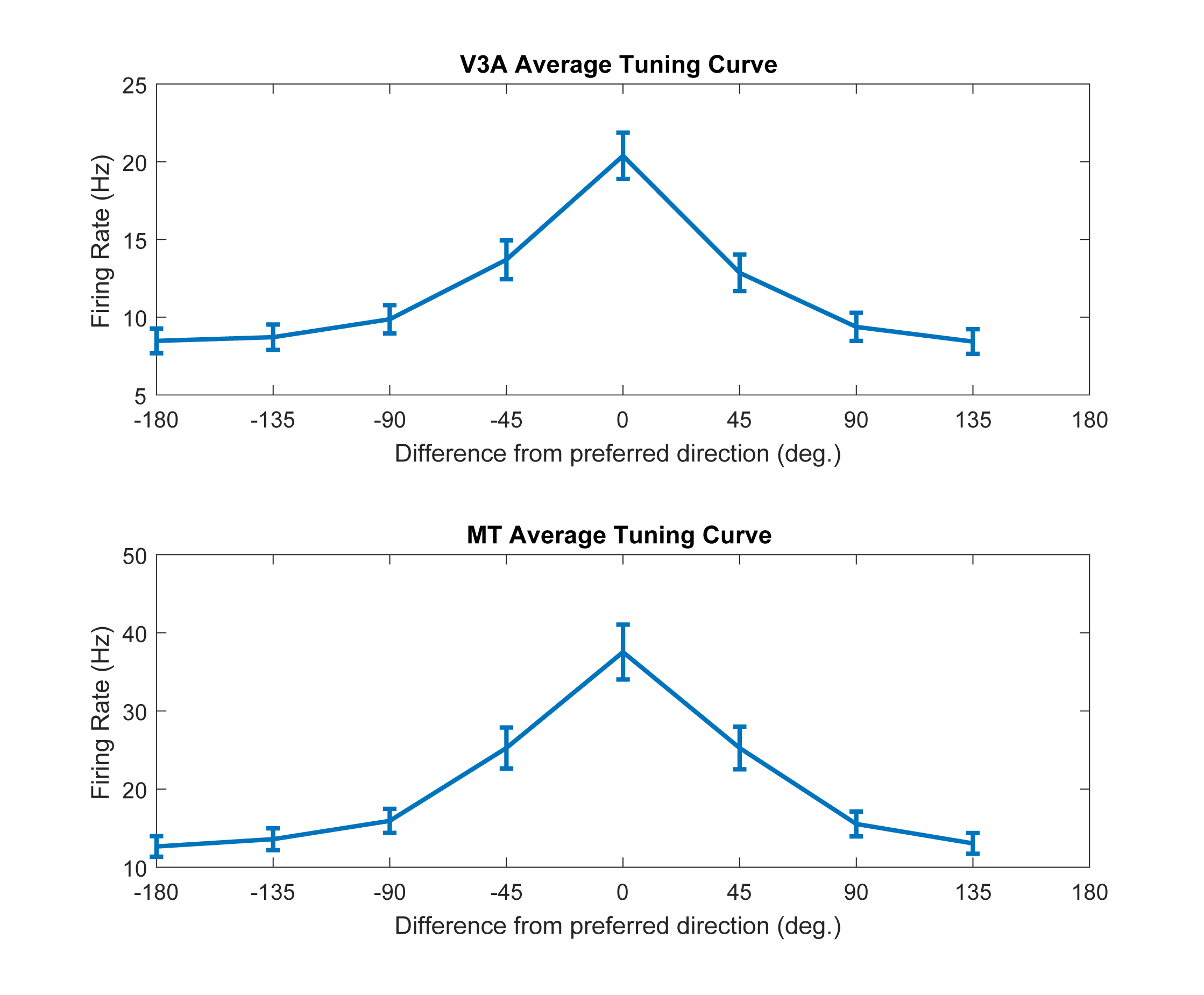

Supplement: Extended Data Figure 6-1 — Average tuning curve for V3A (upper panel) and MT (lower panel). Individual tuning curves are aligned according to the peak firing rate, and then averaged. Download Figure 6-1, TIF file. [file enu-eN-NWR-0383-20-s05.tif]

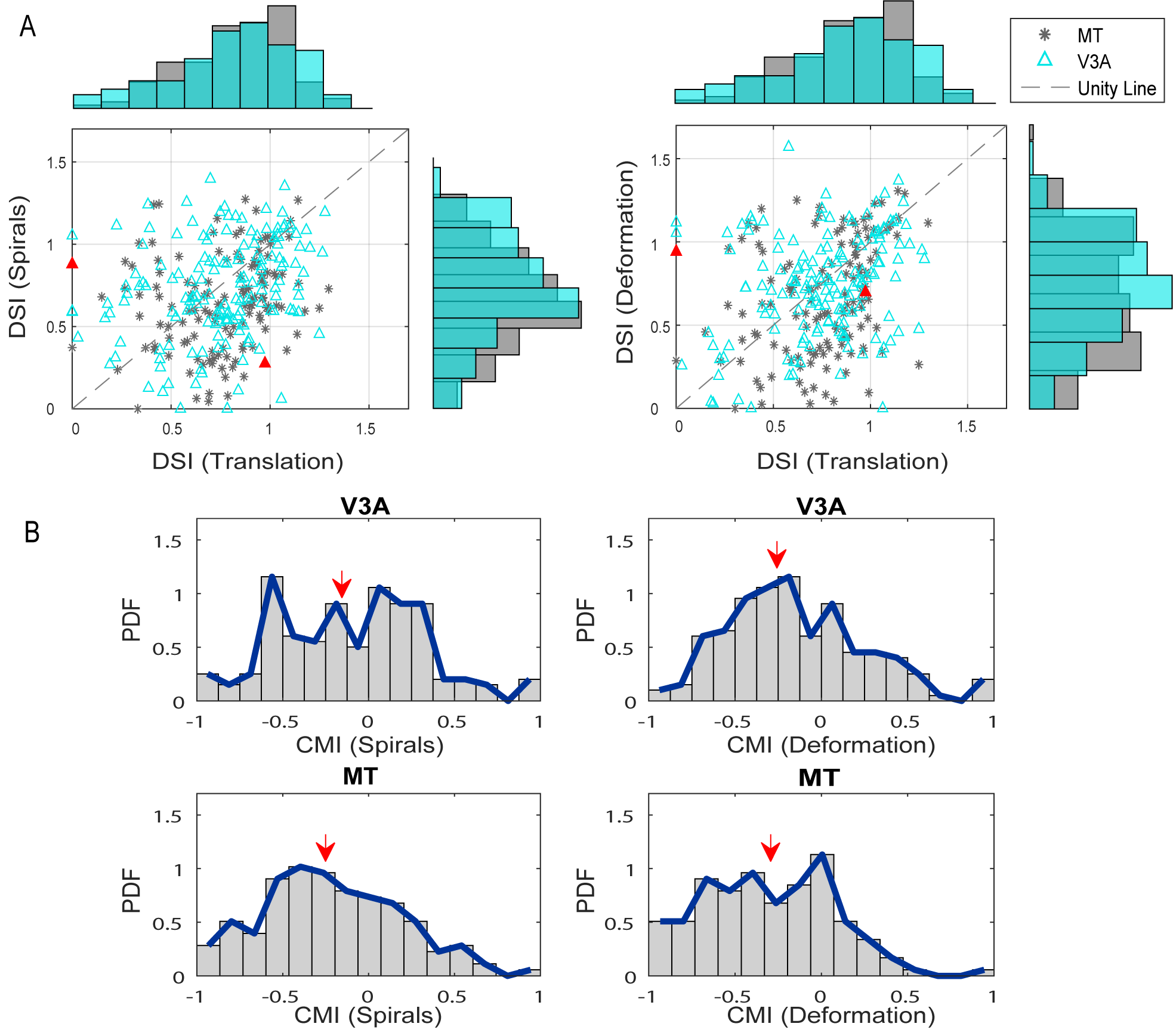

Supplement: Extended Data Figure 7-1 — V3A responses to optic flow stimuli. A, Scatter plots for V3A DSIs (cyan) and MT DSIs (gray) for spirals versus translation (left) and deformation versus translation (right). The unity line is shown by a dashed gray diagonal line. The red triangles refer to the example neurons shown in Figure 6. B, Probability density function of the CMI for sprials (left) and and for deformation (right) for V3A (upper panels) and MT (lower panels). A value of zero for each CMI indicates no preference for complex optic flow over translation motion, while positive numbers indicate a preference for complex optic flow and negative numbers a preference for translation. The red arrows indicate the median for each distribution. For data from each animal shown separately, please see Extended Data Figures 7-1, 7-2. Download Figure 7-1, TIF file. [file enu-eN-NWR-0383-20-s06.tif]

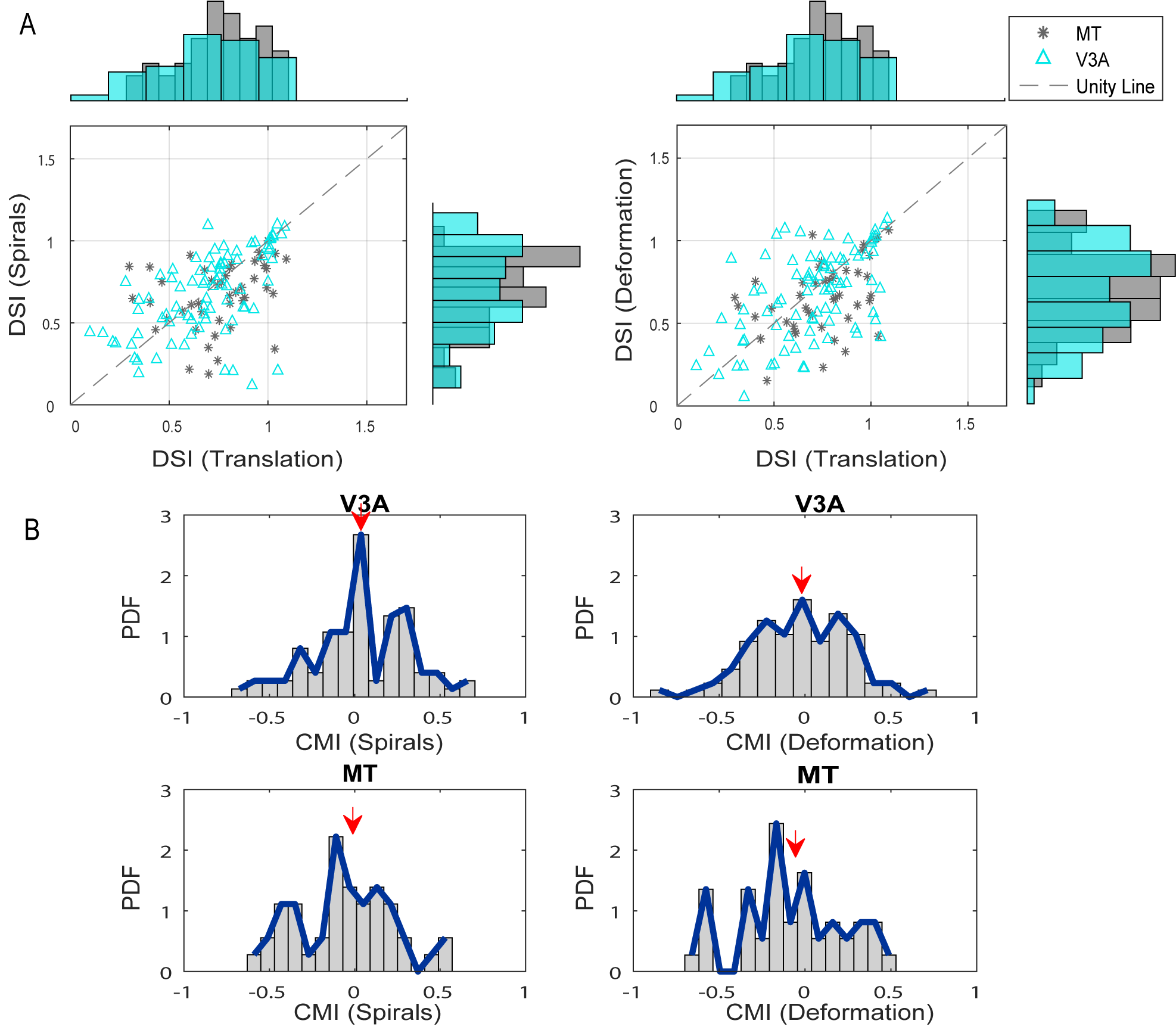

Supplement: Extended Data Figure 7-2 — V3A responses to optic flow stimuli. A, Scatter plots for V3A DSIs (cyan) and MT DSIs (gray) for spirals versus translation (left) and deformation versus translation (right). The unity line is shown by a dashed gray diagonal line. The red triangles refer to the example neurons shown in Figure 6. B, Probability density function of the CMI for sprials (left) and and for deformation (right) for V3A (upper panels) and MT (lower panels). A value of zero for each CMI indicates no preference for complex optic flow over translation motion, while positive numbers indicate a preference for complex optic flow and negative numbers a preference for translation. The red arrows indicate the median for each distribution. For data from each animal shown separately, please see Extended Data Figures 7-1, 7-2. Download Figure 7-2, TIF file. [file enu-eN-NWR-0383-20-s07.tif]
